# Aberrant recruitment of the striatum and insula is associated with recalling and suppressing fatigue- and anger-related memories in people with chronic fatigue syndrome/myalgic encephalomyelitis

**DOI:** 10.1093/braincomms/fcag101

**Published:** 2026-03-26

**Authors:** Katharine A Rimes, Vincent Giampietro, Trudie Chalder, Roland Zahn, Andrew Simmons, Sean James Fallon

**Affiliations:** Psychology and Neuroscience, Institute of Psychiatry, King’s College London, 16 De Crespigny Park, London SE5 8AB, UK; School of Psychology, University of Plymouth, Plymouth PL4 8AA, UK; Psychology and Neuroscience, Institute of Psychiatry, King’s College London, 16 De Crespigny Park, London SE5 8AB, UK; Psychology and Neuroscience, Institute of Psychiatry, King’s College London, 16 De Crespigny Park, London SE5 8AB, UK; Psychology and Neuroscience, Institute of Psychiatry, King’s College London, 16 De Crespigny Park, London SE5 8AB, UK; Psychology and Neuroscience, Institute of Psychiatry, King’s College London, 16 De Crespigny Park, London SE5 8AB, UK; School of Psychology, University of Plymouth, Plymouth PL4 8AA, UK

**Keywords:** striatum, insula, chronic fatigue syndrome/Myalgic encphalomyelitis, functional magnetic resonance imaging, fatigue

## Abstract

Research suggests that people with chronic fatigue syndrome tend to suppress emotions more than healthy individuals. However, whether there are also changes in the neural substrates of emotional regulation in people with chronic fatigue syndrome remain unexplored. Specifically, it is unclear whether there is a neural delineation in how fatigue and anger-related memories are recalled or supressed in people with chronic fatigue syndrome. This study investigated this hypothesis using functional magnetic resonance imaging. We compared blood oxygen level-dependent signal changes between people with chronic fatigue syndrome (N = 20) and matched controls (N = 20) during a novel task that involved the recall or suppression of fatigue (or anger-related memories). The results revealed a dissociation in the contribution of striatal subregions and the insula when recalling and suppressing anger and fatigue-related memories according to diagnostic status. Principally, patients showed higher blood oxygen level-dependent signal in the left and right rostral caudate during the suppression of fatigue and anger-related memories, respectively. Different patterns were also observed in the way each group recruited the posterior putamen when recalling (or suppressing) anger or fatigue-related memories. In contrast to its prominent suppression in striatal regions, blood oxygen level-dependent signal in the insula was increased in the patient group during the *active* recall of anger or fatigue-related memories. Cumulatively, these results reveal that chronic fatigue syndrome is associated with demonstrable, physiological changes in the way emotional information is processed and implicate the rostral caudate and insula as targets for further investigation.

## Teaser

Rimes *et al.* assessed how the neural correlates of suppressing emotional and fatigue-related memories differed between individuals with chronic fatigue syndrome (CFS) and matched controls. In summary, (CFS) individuals showed evidence of increased neural activity in the caudate and insula when memories have to be suppressed or engaged (respectively).

## Graphical Abstract

**Graphical Abstract:**
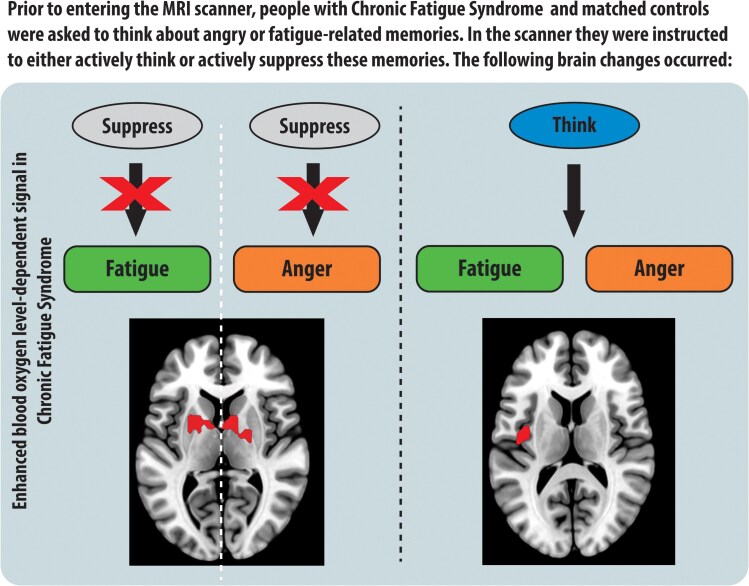

## Introduction

Chronic fatigue syndrome (CFS, sometimes known as myalgic encephalomyelitis ‘ME’) is characterized by excessive mental and physical fatigue of at least six months’ duration, which is associated with substantial impairment in daily activities.^1,2^ This condition is likely to have multiple contributory factors (biological and psychosocial) that promote onset, persistence and influence clinical presentation.^3-5^ One of the neurocognitive factors that has been proposed to contribute to the development or maintenance of this condition is changes in the way emotions are processed.^6,7^ For example, high standards about performance and emotional strength may put individuals at risk of developing chronic fatigue in the context of physical (e.g. a virus) or emotional stressors (negative life events) because they tend to keep pushing themselves to maintain high standards while hiding their stress from others.^8,9^ Consistent with this suggestion, it has been found that people with CFS, compared to controls, have a greater tendency to believe that the experience and expression of negative emotions is unacceptable,^10^ for example, rather than expressing negative emotions in response to failure, there is a tendency to suppress these emotions as failure to meet high standards implies failure as a person. Furthermore, observers rate people with CFS to have lower emotional expression to distressing material than healthy people,^11^ and people with CFS have poorer ability to recognize facial emotions and infer their own emotions.^12^ It is suggested that such emotion processing changes could be one factor contributing to the high comorbidity between CFS and psychiatric conditions.^10,13^ However, the direction of the causal relationship remains unresolved. There are numerous instances where fatigue-like symptoms can precede emotional changes (i.e. depression).^14^ Thus, fatigue may actually antedate emotional changes in CFS. Accordingly, it is possible that emotional processing circuits in CFS/ME may be intact, and that it is predominantly the neurocognitive mechanisms involved in processing fatigue-related information that are impaired.

An alternative hypothesis is that regulation of both fatigue and negatively valenced information (e.g. anger) goes awry through a failure of common cognitive control circuits, that is, there is a common cause that leads to changes in the way fatigue and anger are retrieved or suppressed in CFS/ME. However, it has yet to be firmly established whether individuals with CFS have difficulties in regulating both emotional and fatigue-related information or whether only one of these domains is impaired. A previous fMRI study, which compared the blood oxygenation level-dependent (BOLD) response^15^ in the brain to fatigue and anxiety-related information in CFS and controls, found a substantial inversion in the way these two types of information were processed; CFS patients (compared to controls) displayed increased BOLD signal in posterior parietal and hippocampal regions, but decreased BOLD in dorsolateral/dorsomedial prefrontal cortex when processing fatigue-related information.^16^ This pattern was reversed in anxiety-inducing contexts. Thus, pre-existing evidence suggests a neural origin for differences in the way that negatively valenced and fatigue-related information are processed in CFS. However, there has been a paucity of work investigating the neural correlates of emotion regulation methods or strategies in people with CFS and whether there are distinct regions involved in regulating negative emotions compared to fatigue. Bridging this gap is important as it may be emotional regulation, rather than just mere processing, which may be critically compromised in CFS.^17^

The current study sought to examine whether there were differences in the neural systems used to volitionally engage or suppress negatively valenced or fatigue-related memories. Although there is a need for a synoptic assessment of variations in emotional processes across the spectrum of negative emotions (as in a previous study^11^), we focussed on the control of anger, given the previous findings and that CFS/ME cohorts may have particular difficulty with this emotion.^18^ Participants with CFS and healthy controls were asked to think about or suppress three different types of memories, relating to times when they felt angry, fatigued, or neutral. The main objective was to identify brain areas where people with CFS showed differential activation to healthy individuals when asked to recall or suppress fatigue-related or angry memories. Thus, here, we specifically focus on areas of the brain where the two groups showed differential activation in relation to both thinking strategy (think or suppress) and memory content.

## Materials and methods

### Participants

The study was approved by the Outer East London Research Ethics Committee, Ref 09/H0701/81.

Twenty adults with CFS and 20 healthy adults participated. Sample size was determined on the basis of previous studies that have suggested large effects.^16^ For the CFS group, participants were included if they met Fukuda and Oxford criteria for CFS.^1,2^ Participants were excluded from both groups if they had claustrophobia, any psychiatric condition (assessed using the Mini International Neuropsychiatric Interview;^19^) any metal implants, possible pregnancy or used narcotics, steroids, benzodiazepines or antihypertensives. Participants in both groups were included if they were right-handed with a score of at least 30–100 on the Edinburgh Handedness Inventory.^20^ Healthy participants were selected to be matched for age, gender proportion and education. Healthy participants were excluded if they had a previous diagnosis of CFS or any medical condition likely to cause fatigue. CFS participants were recruited from the South London and Maudsley NHS CFS service after assessment and prior to treatment. Healthy participants were recruited via advertising within King’s College London and through poster advertisements in the local area. The study was conducted before the COVID-19 pandemic.

### Procedure

**Figure 1:**
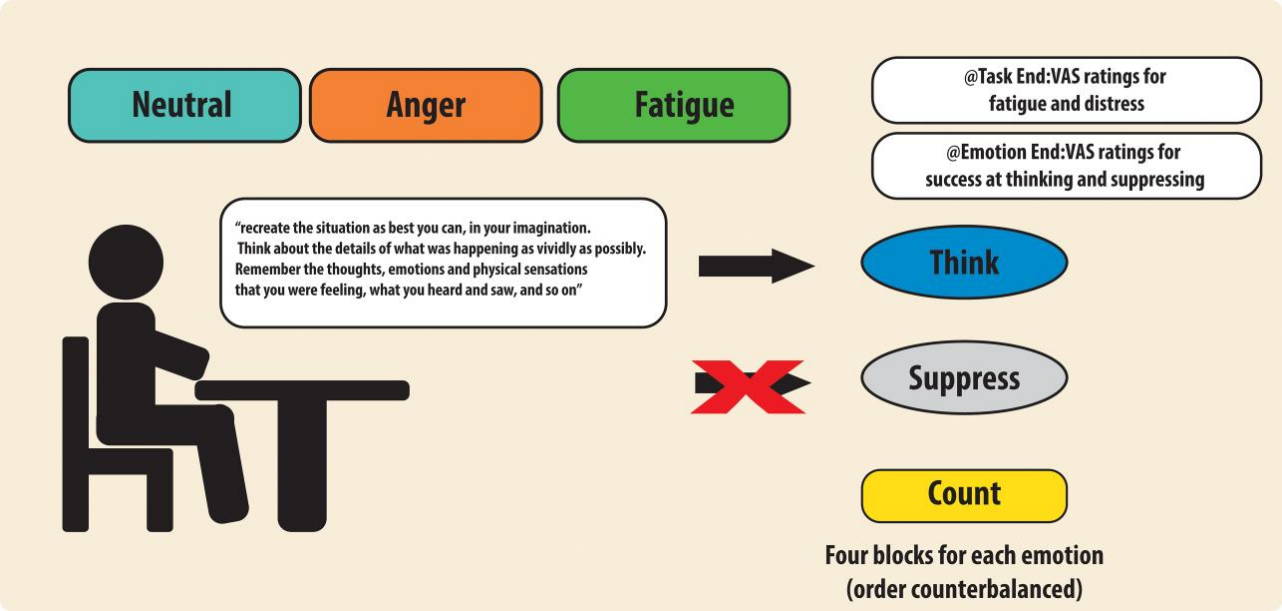
**Summary of key task components**. Briefly, participants were instructed to create three different situations (neutral, angry and fatigue-related). Although in the fMRI scanner, they had to think or suppress this memory when instructed. There were four blocks (think, suppress, count) for each emotion. Ratings of fatigue and distress were collected after each think or suppress condition, whereas success at thinking or suppressing were given after each block.

Participants completed questionnaires prior to coming for their MRI scans. On the day, prior to data acquisition, participants were trained how to respond to three different conditions (Fig. 1): Think, Suppress and Count. For the Think condition, they were asked to ‘recreate the situation as best you can, in your imagination. Think about the details of what was happening as vividly as possibly. Remember the thoughts, emotions and physical sensations that you were feeling, what you heard and saw and so on.’ In the Suppress condition, they were told ‘do not think about the situation in question. Try to suppress thoughts and feelings about it. You can think about whatever else you like, but please try not to think about this situation.’ For the Count condition, they were told to count upwards in their head in English at their own speed. They were given practice at switching between Think, Suppress and Count tasks.

Immediately before the scan, participants were asked to identify three memories. A ‘quiet’ memory was about ‘a situation from the past in which you were sitting quietly, without feeling any strong emotion.’ An ‘anger’ memory was ‘a situation from the past which still makes you feel angry.’ A ‘fatigue’ memory was a memory of a time when they felt extremely fatigued. For each of the three memory types, the three conditions were completed in four blocks; either Think-Suppress-Count, Supress-Count-Think, Count-Suppress-Think, Suppress-Think-Count or Think-Count-Supress, Count-Supress-Think, Supress-Count-Think, and Count-Think-Suppress; participants were randomly allocated to either of these two sets. Within each of the four blocks, Think and Suppress tasks lasted 36 s, and the Count task lasted 22 s. The neutral (quiet) memory set always came first, as this was anticipated to have minimal spill-over effects on subsequent blocks. Participants were randomly allocated to choose anger/fatigue or fatigue/anger for the next sets. Using a joystick, participants completed self-report ratings between tasks and after blocks (described below).

### fMRI image acquisition

Gradient-echo echo-planar MR imaging (EPI) data were acquired at King’s College London, Institute of Psychiatry’s Centre for Neuroimaging Sciences on a General Electric SIGNA HDx 3T MR scanner (GE Healthcare, UK) using the body coil for radio frequency transmission and a quadrature birdcage head coil for reception. In each of 39 noncontiguous planes parallel to the anterior-posterior commissure, 228 T2*-weighted MR images depicting BOLD (blood-oxygen level-dependent) contrasts covering the whole brain were acquired with echo time (TE) = 30 ms, repetition time (TR) = 2 s, flip angle = 75 degrees, in-plane voxel size = 3.75 mm, slice thickness = 3.5 mm, slice skip = 0.5 mm. A high-resolution structural scan (inversion recovery gradient echo planar image) used for standard space normalization of individual activation maps was also acquired in the inter-commissural plane with TE = 30 ms, TR = 3 s, flip angle = 90 degrees, number of slices = 43, slice thickness = 3.0 mm, slice skip = 0.3 mm, in-plane voxel-size = 1.875 mm, providing complete brain coverage.

### Ratings about the scan experience

After each task, participants rated distress and fatigue by moving a joystick to indicate responses on a 0–100 visual analog scale (VAS). At the end of each set (neutral, anger, or fatigue), participants rated their success at suppression in the Don’t Think blocks and percentage of time thinking of the situation during the Think and Don’t Think blocks. After the scan, participants were asked to rate memory vividness, suppression effort and percentage of time spent counting in the count tasks. Finally, postscan, participants were asked about the memories that they were focusing on in each condition. The memories participants reported were rated by our research team as adhering to the instructions given, that is, angry memories were angry and fatigue-related memories were fatiguing.

### Questionnaire measures

Participants completed the following: the Chalder Fatigue Scale to assess fatigue.^21(p93)^ In completing the Chalder Fatigue Scale, they were asked the following: ‘We would like to know more about any problems you have had with feeling tired, weak or lacking in energy in the last month? If you have been feeling tired for a long time, please compare yourself to how you felt when last well.’ Thus, participants were asked to rate their symptoms within the last month or since they were last feeling well. It should be noted that the minimal Chalder Fatigue Scale score is 11. Physical Functioning subscale of the SF-36^22^ to assess physical functioning; Work and Social Adjustment Scale^23^ to assess impairment in daily activities and Hospital Anxiety and Depression Scale^24^ to assess anxiety and depression.

### Data preparation and analysis

#### Behavioural analysis

Behavioural data were analysed using JASP(0.19.3).^25^ Comparable nonparametric ANOVAs were performed using R (3.6.3) in RStudio (1.2.5003). We used the Aligned rank transform package^26^ to perform nonparametric ANOVAs.

#### Functional imaging data

Functional imaging data were analysed using XBAM version 4.1, an fMRI analysis software package developed at King’s College London’s Institute of Psychiatry, Psychology & Neuroscience,^27^ that uses a nonparametric approach to minimize assumptions.^28^ The software and methods have been used in over 200 peer-reviewed publications.^29,30^

Data were first processed to minimize motion-related artefacts.^31^ Following realignment, images were smoothed using an 8.83 mm full-width half-maximum Gaussian filter. Responses to each condition were then detected by time-series analysis using a linear model in which each component of the experimental design was convolved separately with a pair of Poisson kernels (λ= 4 and 8 s) to allow variability in the haemodynamic delay. The best fit between the weighted sum of these convolutions and the time-series at each voxel was computed using a constrained BOLD effect model.^32^ In each experimental task, the Count condition was contrasted to the Think and Suppress conditions. A goodness-of-fit statistic was then computed as the ratio of the sum of squares of deviations from the mean image intensity resulting from the model (over the whole time-series) to the sum of squares of deviations resulting from the residuals (SSQ ratio).

Following the computation of the observed SSQ ratio at each voxel, the data were permuted by the wavelet-based method.^33^ The observed and permuted SSQ ratio maps for each individual were transformed into the standard space of Talairach *et al*.,^34^ using a two-stage warping procedure).^27^ Group maps of activated voxels were then computed using the median SSQ ratio at each voxel (over all individuals) in the observed and permuted data maps.^27^ Computing intra- and inter-participant variations in effect separately constitutes a mixed effect approach, which is desirable in fMRI. Detection of activated regions was extended from voxel to 3D cluster-level using the method.^35^

The interaction between group membership (patients and controls), experimental condition (neutral, emotional, and fatigue) and experimental task (think and suppress) was determined using a split-plot analysis of variance (ANOVA) model. The model is fitted by minimizing the sum of absolute deviations to reduce outlier effects. The null distribution is computed by permuting data between groups/tasks/conditions and refitting the model a maximum of 50 times at each voxel and combining the data over all intracerebral voxels. Using the derived null distributions, all resulting 3D cluster-level maps were thresholding in such a way as to yielding less than one expected Type I error cluster per map.

#### Voxel-based morphometry (VBM**)** analysis

To examine whether structural differences could contribute to functional differences, we also examined brain structure. Structural differences between groups were evaluated using a unified method of voxel-based morphometry (VBM) within statistical parametric mapping software version 8 (SPM-8) (Wellcome Department of Cognitive Neurology, Institute of Neurology, London, UK; http://www.fil.ion.ucl.ac.uk/spm/software/spm8/). Initially, T1-weighted images for each subject were manually oriented to place the anterior commissure and posterior commissure in alignment with the Montreal Neurologic Institute (MNI) co-ordinate system. Images were then normalized into standard anatomic space using a linear 12-parameter affine transformation with the SPM-8 default MNI template as reference. An integrated modulation step was applied in conjunction with the normalization procedure in order to correct for any expansions/contractions in brain size, and to preserve grey and white matter volumes in each voxel. Normalized images were then segmented into grey matter (GM) and white matter (WM) using a mixed-model cluster analysis technique that uses prior probability maps to classify tissue types within each voxel.^36^ Segmented images were then smoothed with an 8 mm full-width at half-maximum isotropic Gaussian kernel in order to reduce differences caused by variations in individual grey and white matter structure. Finally, all images were visually inspected for registration errors. We examined evidence for regionally specific (voxel-wise) grey matter differences between the two groups (CFS and controls) using the general linear model.

### Data availability

Our ethics approval does not allow public archiving of the data used in this study. Those wishing to access to these data should contact the lead author, KAR. Access can be obtained and granted to named individuals. To obtain the data, investigators would need to complete a formal data sharing agreement, including conditions for secure storage of sensitive data.

### Results

#### Participant characteristics

Participant sociodemographic, clinical and suppression-related characteristics are shown in Table 1. The mean illness duration for the participants with CFS was 1.3 years (SD 2.2). None of the patients was taking medication explicitly for their Chronic Fatigue. With regards to pain medication, 4 (out of 20) patients were taking pain relief medications (opioids and/or nonsteroidal anti-inflammatory drugs; NSAIDs).

**Table 1:** Comparison between participants with CFS and healthy controls on demographic and clinical variables

	Participants with CFS (n = 20)	Healthy Controls (n = 20)	
% women	40%	40%	(χ^2^(_1,_) = 1.0, *P* = 1.0
Education beyond age 16	75%	75%	(χ^2^(_1_ (_1,_) = 1.0, *P* = 1.0
Ethnicity: proportion non-white	10%	20%	Fisher’s exact test = 0.66
Age (years)	Mean (SD) 32.0 (11.8)	Mean (SD) 30.3 (9.3)	t(38) = 0.5, *P* = 0.62
Chalder Fatigue Scale	25.7 (4.7)	10.0 (1.9)	t(38) = 13.7, −*P* < 0.0005
Work and Social Adjustment Scale	26.3 (7.8)	1.0 (4.5)	t(38) = 12.6, *P* < 0.0005
Physical Functioning Scale, SF36	61.4 (21.7)	99.0 (2.8)	t(38)=−7.6, *P* < 0.0005
Hospital Anxiety and Depression Scale			
‒ Depression	6.8 (2.6)	0.9 (1.2)	t(38) = 9.3, *P* < 0.0005
‒ Anxiety	7.7 (4.8)	4.1 (2.4)	t(38) = 3.0, *P* = 0.005
Condition Duration	1.3 (2.2)	NA	
Age at symptom onset	29.8 (11)	NA	
Not in work/studying	3/20	0/20	(χ^2^(_1_) = 3.24, *P* = 0.07)
Hours of Paid Work	21.62 (16.8)	29.1 (14)	*t*(38) = 1.5, *P* = 0.13

#### Behavioural performance

##### Self-reported task performance

First, we sought to determine whether our manipulations were successful and whether there were differences in task adherence between groups. The groups were compared on self-reported task performance. Independent t-tests indicated no significant group differences on postscan ratings of vividness of any of the three memories, efforts to suppress in the three conditions or the percentage of time spent counting in the counting condition (t values < −1.7). Perceived success at suppression for the suppression tasks was investigated using a 2 * 3 (Group: CFS, HC; Condition: neutral, fatigue and anger) repeated measures ANOVA. This indicated no significant effects of group, condition or group by condition interaction (F values < 2.3). Self-reported percentage of time spent thinking about the memory was investigated using a 2 * 3 * 2 (Group: CFS, HC; Condition: neutral, fatigue, anger; Task: think, suppress) repeated measures ANOVA. Data from three patients and one control were missing due to technical issues. There was a significant effect of task (F(1,34) = 279.2, *P* < 0.00001, η_p_^2^ = 0.891) with a greater percentage, thinking about the memory in the Think tasks than in the Suppress tasks. There were no other significant main effects or interactions (all p’s > 0.11). Thus, cumulatively, evidence from self-ratings indicates that the task manipulation worked, with no evidence that the groups differed in their subjective ability to adhere to the task instructions.

##### Effect of the task on self-reported ratings of fatigue

Mean fatigue ratings taken in the scanner, immediately after each task, were investigated using a 2 * 3 * 2 repeated measures ANOVA (Group: HC, HC; Condition: Neutral, Fatigue, Anger; Task: Think, Suppress; Table 2). As expected, this indicated a main effect of group (F(1,38) = 19.3, *P* = 0.00008, η_p_^2^ = 0.33) with the CFS group reporting higher ratings of fatigue than the HC across the tasks. There was a significant main effect of condition (F(2,76) = 17.28, *P* < 0.00001, η_p_^2^ = 0.31) and task (F(1,38) = 18.5, *P* = 0.0001, η_p_^2^ = 0.32), and these were modified by a significant condition by task interaction (F(2,76)= 9.89, *P* = 0.00015, η_p_^2^ = 0.32). There was evidence for a significant three-way interaction (F(2, 76) = 3.56, *P* = 0.033, η_p_^2^ = 0.086). This interaction generally results from a tendency for differences between-group differences in fatigue in the think conditions (Condition × Group interaction for Think trials; F(2,76) = 2.64, *P* = 0.078, η_p_^2^ = 0.065), without any evidence for such an effect in the suppress conditions (Condition × Group interaction for Suppress Trials; (F < 1)). There were no other significant interactions (all *P* > 0.21).

**Table 2:** Mean post-task ratings of fatigue and distress

	CFS (n = 20)		HC (n = 20)	
Condition/Task	Mean	SD	Mean	SD
	Fatigue (0–100 rating scale)			
Neutral/Think	42.3	(31.2)	6.1	(11.2)
Neutral/Suppress	38.0	(31.6)	6.8	(12.3)
Fatigue/Think	59.3	(28.9)	39.4	(32.1)
Fatigue/Suppress	51.1	(29.7)	20.9	(26.8)
Anger/Think	50.1	(31.6)	15.4	(21.3)
Anger/Suppress	48.7	(33.1)	10.9	(17.5)
	Distress (0–100 rating scale)			
Neutral/Think	15.3	(16.4)	2.5	(5.1)
Neutral/Suppress	13.0	(12.9)	3.4	(6.6)
Fatigue/Think	26.6	(22.9)	19.3	(29.1)
Fatigue/Suppress	19.0	(19.6)	5.3	(7.6)
Anger/Think	38.7	(27.9)	30.0	(34.2)
Anger/Suppress	24.3	(23.3)	10.2	21.7

*Effects of the task on self-reported ratings of distress:* A 2 * 3 * 2 repeated measures ANOVA (Group: HC, HC; Condition: Neutral, Fatigue, Anger; Task: Think, Suppress) was undertaken to compare the two groups on mean distress ratings taken in the scan immediately after each task (Table 2). This indicated a main effect of group (F(1,38) = 5.3, *P* = 0.027, η_p_^2^ = 0.12) with the CFS having higher ratings of distress than the HC. Significant main effects were observed for condition (F(2,76) = 14.16, *P* < 0.00001, η_p_^2^ = 0.27) and Task (F(1,38) = 20.8, *P* = 0.00005, η_p_^2^ = 0.35), and these were modified by a significant Condition by Task interaction (F(2,76) = 17.2, *P* < 0.00001, η_p_^2^ = 0.31). Simple-main effects analyses revealed that there was no evidence for a difference in distress ratings after completing think versus suppress trials on neutral memory (F < 1). However, there was significantly greater distress for thinking compared to suppressing in the anger (F(1,38) = 27.25, *P* < 0.000001, η_p_^2^ = 0.41) and thinking versus suppressing in the fatigue condition (F(1,38) = 12.72, *P* = 0.0009, η_p_^2^ = 0.25). There were no other significant interactions (p’s > 0.17).

Equivalent, nonparametric ANOVAs yielded corroborating results for all the above analyses.

#### Neuroimaging Analysis

##### CFS patients differ in the extent to which striatal and insula regions are recruited

**Figure 2:**
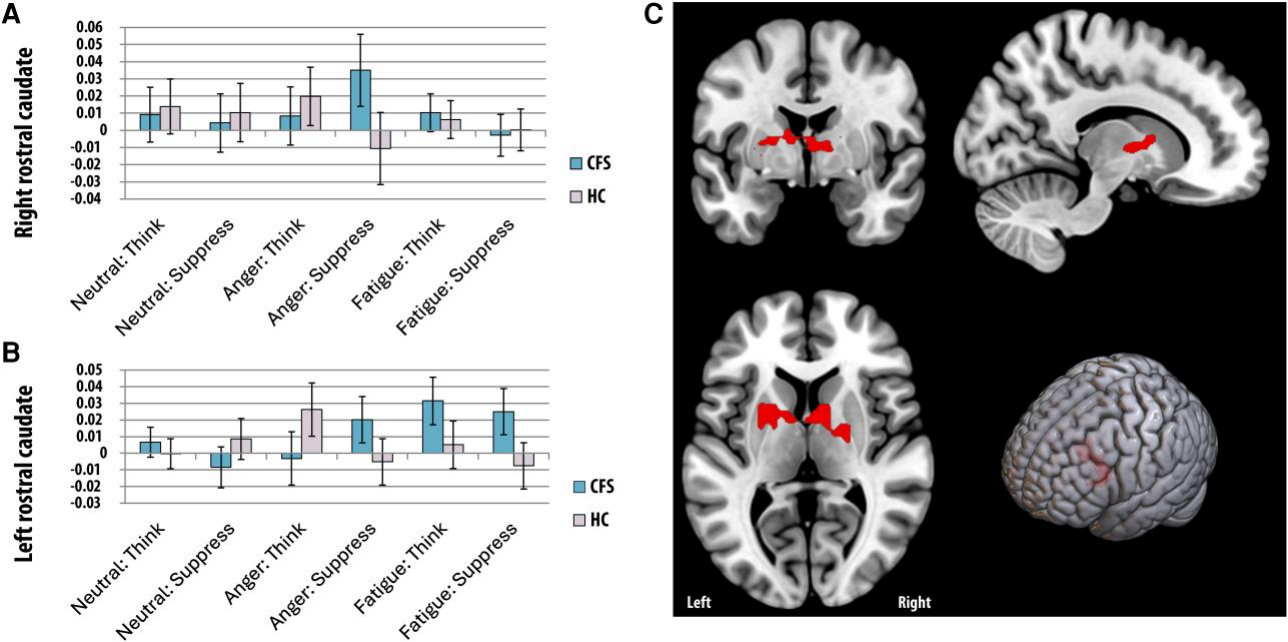
**BOLD signal changes in the rostral caudate.** Differences in right caudate (peak at 11,8,1, Panel **A**) and left caudate (peak at −12,9,0, Panel **B**) between people with chronic fatigue syndrome (CFS) and controls across different tasks (means and standard errors). Coronal, Sagittal, horizontal and 3D depictions of significant clusters in the left and right rostral caudate (cluster-based permutation test, *P* < 0.05, whole-brain corrected, Panel **C**).

**Figure 3:**
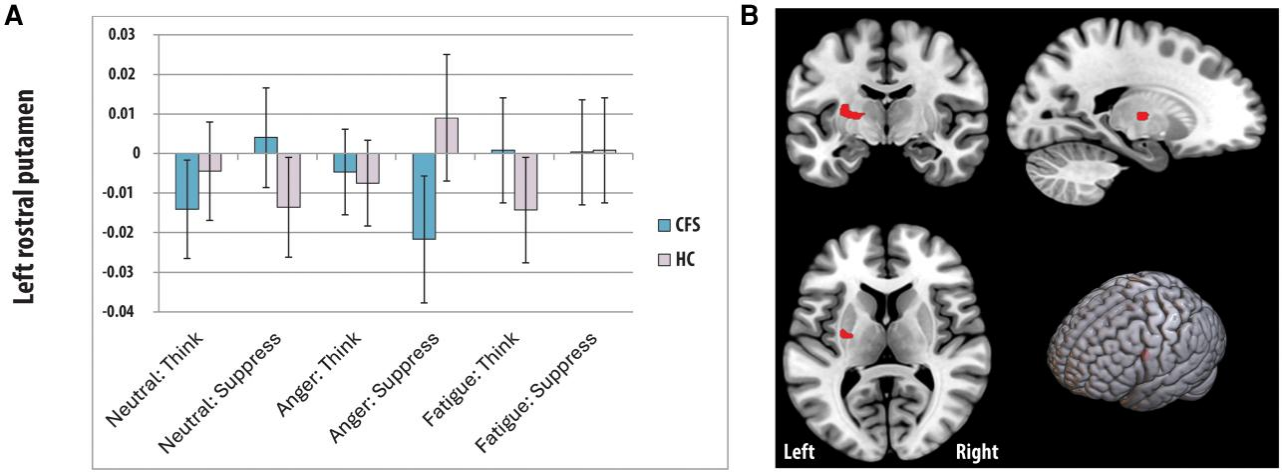
**BOLD signal changes in the posterior putamen.** Differences in left putamen activation (peak at −31, −14, 2, Panel A) between people with chronic fatigue syndrome (CFS) and controls across different tasks (means and standard errors). Coronal, Sagittal, horizontal and 3D depictions of significant cluster within the posterior putamen (cluster-based permutation test, *P* < 0.05, whole-brain corrected, Panel B).

**Figure 4:**
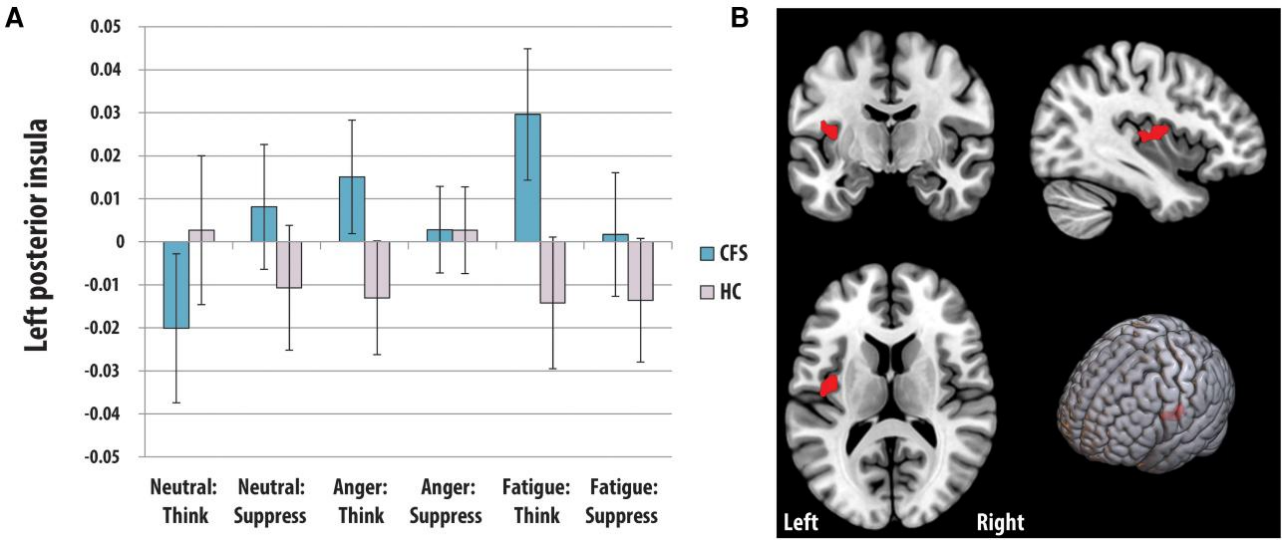
**BOLD signal changes in the left anterior insula.** Differences in left posterior insula activation (peak at −40, −8, 17, Panel A) between people with chronic fatigue syndrome (CFS) and controls across different tasks (means and standard errors). Coronal, Sagittal, horizontal and 3D depictions of significant cluster within left posterior insula (cluster-based permutation test, *P* < 0.05, whole-brain corrected, Panel B).

A whole brain, group (CFS, control) × emotion (anger, fatigue) × task (neutral, think, suppress) ANOVA showed four significant three-way interaction clusters (Figs 2 to 4). **Cluster 1** was in the right rostral caudate extending into the putamen and thalamus; 3D cluster size = 48, peak Talairach co-ordinates (x,y,z) = 11,8,1; corrected cluster *P* value =0.014. **Cluster 2** was in the left rostral caudate; 3D cluster size = 60, peak Talairach co-ordinates = −12, 9, 9; corrected cluster *P* value =0.006. **Cluster 3** was in the left posterior putamen extending into the thalamus; 3D cluster size = 49, peak Talairach co-ordinates = −31, −14, 2; corrected cluster *P* value =0.016. **Cluster 4** was in the left posterior insula (Brodman Area 13); 3D cluster size = 18, peak Talairach co-ordinates = −40, −8, 17; corrected cluster *P* value =0.015. The underlying average statistical values (SSQs) were extracted for each cluster and for each subject, followed by post-hoc comparisons. The findings are summarized below.

##### Right rostral caudate is differentially recruited by CFS and controls according to task context (cluster 1)

Between groups, the CFS group, compared to controls, had higher BOLD signal in the right rostral caudate during the suppression of anger (Fig. 2A; *P* = 0.036). There was no evidence for differences in the other conditions. Within groups, controls showed increased BOLD signal in this region when thinking about anger-related memories compared to suppressing them (*P* = 0.006). In contrast, CFS group showed a nonsignificant trend for a higher BOLD signal during suppression than thinking about anger-related memories (*P* = 0.052).

##### CFS participants show signal BOLD signal in the left rostral caudate when suppressing fatigue-related memories (cluster 2)

Contrasting groups’ differences in cluster 3 (left rostral caudate), CFS participants displayed higher BOLD signal during fatigue suppression than controls (Fig. 2B, *P* = 0.026); similar nonsignificant trends were found for thinking about fatigue (*P* = 0.074) and suppressing anger (*P* = 0.080). In contrast, there was a nonsignificant trend for lower CFS participants to have lower BOLD signal in the left rostral caudate when thinking about anger (*P* = 0.073). Within the CFS group, pairwise comparisons indicated that Think Fatigue tasks had significantly higher BOLD signal than Think Anger, Think Neutral and Suppress Neutral scenarios (*P* values <0.05). Controls, however, showed evidence for increased BOLD signal when thinking about anger compared to all other conditions except Suppress Neutral.

##### Distinct patterns of left posterior putamen recruitment are observed between CFS and controls (cluster 3)

Regarding differences in the posterior putamen (Fig. 3), there was only a trend for lower BOLD signal in the CFS group, compared to the control group, during the suppression of anger (*P* = 0.065). However, there was evidence that the BOLD signal in the posterior putamen differed between groups according to task context. Controls showed higher BOLD signals during the suppression of anger compared to thinking about fatigue (*P* = 0.027). This pattern was not evident in CFS, where the suppression of anger led to significantly lower BOLD signal than the suppression of neutral memories (*P* = 0.047).

##### CFS participants show increased BOLD signal in the left insula when thinking about anger and fatigue (cluster 4)

Comparing groups revealed significantly higher BOLD signal in the left insula for the CFS (Fig. 4), compared controls, for both thinking about anger (*P* = 0.039) and fatigue (*P* = 0.007). Within the groups, CFS patients showed lower BOLD signal during thinking about neutral memories compared to thinking about anger, thinking about fatigue and suppressing neutral memories (*P* values < 0.05). In the HC groups, BOLD differences were only observed between thinking about anger and suppressing anger (*P* = 0.006).

**Structural Brain Analyses:** There was no evidence for differences in grey matter (no significant whole-brain voxel-wise differences were observed between the CFS and HC groups).

*Exploratory BOLD-behavioural analyses:* To contextualize the above effects, a series of exploratory correlations was performed to examine the relationship between the BOLD signal increases and self-report measures.

First, we examined the relationship between right rostral caudate BOLD signal when suppressing anger and self-report measures (distress and fatigue). There was no strong evidence for an association between BOLD signal in this region and these self-report measures. For example, within the healthy participants, correlational analyses indicated that right caudate activation in the Suppress Anger condition was not significantly associated with post-scenario distress (r(20) = 0.05, *P* = 0.83); the association with fatigue showed a non-significant trend (r(20) = −0.43, *P* = 0.061). For the CFS group, there was no significant association between right caudate activation in the Suppress Anger condition and ratings of fatigue (r(20) = − 0.35, *P* = 0.13) or distress (r(20) = −0.28, *P* = 0.24). For both groups combined, none of these associations was significant.

We then examined the relationship between the BOLD signal in the left rostral caudate during the suppression of fatigue and self-report measures (distress and fatigue). These analyses revealed evidence for different relationships between the BOLD signal and behaviour according to group. In controls, there was a significant association between left caudate activation in the Suppress Fatigue tasks and post-scenario fatigue ratings (r(20) = 0.63, *P* = 0.003) but not with distress (r(20) = 0.18, *P* = 0.45). For the CFS group, there was no significant association between left caudate activation and post-task ratings of fatigue (r(20) = −0.18, *P* = 0.46) or distress (r(20) = 0.04, *P* = 0.87). For both groups combined, no correlations were significant.

Turning to the left posterior putamen, we investigated how the BOLD signal during the suppression of anger related to both self-report measures (distress and fatigue). These analyses revealed no evidence for an association. For the HC group, left putamen activation in the Anger Suppress tasks was significantly associated with lower fatigue (r(20) = −0.50, *P* = 0.024), but the association with distress was not significant (r(20) = −0.345, *P* = 0.137). For the CFS, there was no significant association between left putamen activation in Anger Suppress and fatigue (r(20) = 0.15, *P* = 0.53) or distress (r(20) = 0.04, *P* = 0.86). When both groups were combined, the associations with fatigue and distress were not significant.

Next, the relationship between self-reported anger/fatigue and BOLD signal in the left insula during thinking about the anger task was examined. Both groups showed evidence for an association between insula BOLD signal and distress but not fatigue, i.e. *there was a significant association between left insula activation in the Think Anger tasks and post-scenario ratings of distress (r(40)=−*0*.410, P* = *0.009) but not fatigue (r(40)=−*0*.11, P* = 0*.51).* Breaking this down, Controls showed that a greater left insula BOLD signal was significantly associated with lower post-scenario distress ratings (r(20)=−0.62, *P* = 0.004); the association with fatigue was not significant (r(20) = −0.314, *P* = 0.18). In the CFS group, there were nonsignificant trends for left insula BOLD signal when thinking about anger to be associated with lower distress (r(20) = −0.441, *P* = 0.052) and lower fatigue (r(20) = −)0.39, *P* = 0.092).

Finally, we examined the relationship between the left insula BOLD signal during thinking about fatigue and self-report measures (distress and fatigue). There were no significant associations left insula BOLD (thinking about fatigue) or distress in controls (postscenario fatigue (r(20) = −0.13, *P* = 0.58; distress (r(20) = 0.29, *P* = 0.21) or in the CFS (left insula, thinking about fatigue): postscenario fatigue (r(20) = 0.03, *P* = 0.92); distress (r(20) = 0.6, *P* = 0.80).

### Discussion

This is the first experimental fMRI study to investigate neural correlates of recall and suppression of fatigue and anger-related memories in people with CFS compared to well-matched healthy individuals. Behaviourally, both groups had comparable performance on the task, that is, there were no significant group differences in self-reported effort to suppress the memories, success at suppression, time spent thinking about the memories or memory vividness. However, there was evidence for substantial group differences in the neural correlates of performing these tasks. The rostral caudate indicated a group by emotion interaction; areas of the left and right rostral caudate were disproportionately recruited by CFS patients during the suppression of fatigue and anger-related memories, respectively. These patterns were not seen across other striatal subregions, such as the posterior putamen. Although there was mostly evidence for exaggerated striatal recruitment during the suppression of memories in CFS, the general trend in the insula was for increased BOLD signal during the active recall of anger and fatigue-related memories. Thus, both striatal and insula aberrations appear to co-exist in CFS participants. Cumulatively, these data suggest that the neural origin of emotional dysregulation in CFS is multifactorial, comprises multiple neural systems and is highly task-dependent.

#### Hemispheric division of labour in the caudate in CFS

Our analyses showed a three-way interaction between group (CFS, Control), emotional category (Anger, Fatigue) and regulation (think or suppress) with separate clusters appearing in the left and right rostral caudate. Breaking down this interaction revealed different patterns of neural modulation according to group. CFS participants showed higher BOLD signal in the *left* rostral caudate for suppressing fatigue-related memories, but higher BOLD signal in the *right* when suppressing anger-related memories. Although there is some evidence for lateralized inhibition networks in the general population,^37^ no such discrepancy between the way the left and right rostral caudate regions process suppress anger versus fatigue-related information was evident in controls (Fig. 2). This suggests that there is a hemispheric component to neuronal dysregulation in CFS within the rostral caudate region.

The association of the rostral caudate with suppression is unsurprising, given the long tradition of associating this region, in conjunction with the prefrontal cortex, with maintaining normal behavioural control, particularly with regard to formulating alternatives to to-be-suppressed or to-be-ignored information.^38-40^ Recent meta-analyses corroborate this view as the caudate has been found to be particularly active during the withholding rather than cancelling certain behaviours.^41^ Therefore, the exaggerated neuronal responses in the caudate during suppression in CFS are consistent with the known neural substrates of this function.

With regard to the lateralized nature of the effects, reports of hemispheric discrepancies in CFS are not unprecedented. Indeed, of the relatively few fMRI studies in CFS, deficient neuronal activation was found to be strongly lateralized in the basal ganglia.^42^ Decreased BOLD signal in the right caudate during the response to winning (compared to losing) was observed in CFS participants, that is, relatively higher BOLD signal in CFS, compared to controls, was found for negative compared to positive information in the right caudate. Thus, the results from the previous study concords well with the present study, in that both studies found relatively increased BOLD signal in the right caudate when processing negative information.

The causes of this lateralization of emotional circuitry in CFS remain unknown, but numerous testable hypotheses present themselves. One of the most likely culprits for these lateralized effects is changes in dopaminergic stimulation, either directly or vicariously through dopamine-immune system interactions.^42^ Redolent neural changes have frequently been observed in dopamine-related disorders such as Parkinson’s disease (PD). For example, evidence from a positron emission tomography study suggests a prominent role for left caudate in driving fatigue in PD.^43^ Meta-analyses have also demonstrated reliable lateralization of dysfunction in the caudate in patients with depression.^44^ Therefore, lateralization within the caudate appears to be a feature of many neuropsychiatric conditions, which might indicate that a causal role for disruption to neurotransmitter systems in promoting neural changes in CFS. Future neuropsychopharmacological studies should be conducted to test these hypotheses, for example, dopaminergic drugs that can readily alter neuronal processing within the striatum.^45^ Teasing apart the hemispheric contributions of left versus right striatum could also be achieved with noninvasive transcranial ultrasound.^46^

#### Different patterns of left posterior putamen in CFS and controls

In distinction to the above BOLD signal changes in the caudate, we observed very different patterns in a separate area of the striatum: the left posterior putamen. A previous study in healthy individuals reported that the instruction to plan to suppress emotions resulted in increased activation in the bilateral putamen, which was not observed in the instruction to prepare for cognitive appraisal.^47^ In that study, the degree of activation predicted lower motor-related activity in the precentral gyrus during subsequent action suppression. The authors interpreted their findings as suggesting emotional suppression is associated with enhanced activation in the putamen, which, in turn, contributes to the reduced facial emotional expression. With regard to CFS, clinical reports^9^ and an experimental study^11^ suggest reduced facial emotional expression. Thus, together with the present findings, there may be a key role for posterior putamenal activation changes in contributing to emotional changes in CFS. The relationship between left putamen activation during suppression and fatigue warrants further investigation in both healthy individuals and those with CFS.

#### Increased insula BOLD signal when actively thinking about anger and fatigue in CFS patients

The striatum receives input from the insula, which was also implicated in the present study. Although long thought of as primarily involved in mediating gustatory responses, the insula plays a prominent role in shaping our cognitive and emotional experience.^48,49,50^ Here, the posterior insula was found to be hyperactive in CFS patients when they had to think about anger and fatigue-related memories. Thus, although there were no differences in the self-reported measures used to assess task compliance, this suggests that the neurocognitive mechanisms used to support this process in CFS may be distinct from those in controls. The insula’s involvement in the intentional regulation of physiological arousal,^51^ and the heightened physiological arousal present in CFS,^52^ may implicate this region as one of the neural loci responsible for maintaining CFS symptoms.^53^ Congruent with this view that it could also be the case that the neural source for the habitual, recalcitrant persistence of CFS symptoms resides within the insula. For example, insula dysfunction is also a feature of addiction,^54-56^ particularly in terms of driving cue reactivity and cravings.^57^ It should be noted, however, that the functions of the insula are diverse; insula activation drives cognitive shifts^58^ and a specific role for the left insula in executive shifting has been proposed.^59^ Again, such accounts remain speculative, and future therapeutic interventions in CFS may want to explore these hypotheses by modulating the activity of the insula, for example, with low-intensity focused ultrasound^60^ or transcranial temporal interference stimulation (tTIS^61^).

#### No structural brain differences were observed between patients and controls

Here, the principal aim of performing structural analyses was to examine whether there were any large differences in brain structure that might contribute to the observed functional changes in CFS. We did not find any evidence to support this claim. It could be argued that our study is underpowered to detect any differences. However, our findings are in line with large, well-powered studies^62^ (although see^63^ for a review and alternative findings). Thus, although the existence of structural brain changes in CFS as a population remains an open question, it seems unlikely that structural differences between our groups could have contributed to the BOLD signal changes we report.

#### Limitations of the study

Although participants from both groups were excluded if they had current psychiatric problems, the CFS participants had significantly higher self-reported anxiety and depression symptoms. This is consistent with the robust evidence of an association between CFS and depression and anxiety.^64,65^ The nature of the relationship is unclear, but many of the contributory factors suggested for CFS are also implicated in psychiatric disorders, such as childhood trauma, unhelpful beliefs and HPA axis disturbance. Therefore, controlling for depression and anxiety may obscure rather than improve our understanding of this condition. Attempting to recruit a CFS sample matched for levels of anxiety and depression would result in a sample that would be highly atypical of people with CFS who are seeking treatment. Studies such as the present one may help improve our understanding of common mechanisms between depression/anxiety and CFS, as the brain areas identified here have all been implicated in depression and anxiety, too. Finally, this study did not capture measures of circadian rhythms in people with CFS. Although subtle, there may be sleep changes in people with CFS that may contribute to fatigue.^66^ Future studies should incorporate direct measures of sleep quality in people with CFS.

### Conclusions

The findings suggest that there are distinct neural correlates of alterations in the processing of fatigue and emotion stimuli in people with CFS, which are not underpinned by structural differences.

The results illustrate the complexity of the neural changes that support emotional processing in CFS; neural changes are not circumscribed to one neural locus but are distributed across subcortical and paralimbic areas. Principally, there was evidence for a higher BOLD signal within the rostral caudate during the suppression of fatigue- and anger-related memories. In contrast, there was a lower BOLD signal in the insula during the active recall of these memories. The present results suggest that normalizing emotional processing circuits may require upregulation of insula regions, but downregulation of striatal regions. Future studies should look at whether this can be achieved through neuromodulation in these regions^67^ or through psychological interventions, for example, cognitive behavioural therapy (CBT)^68^ and connectivity with frontal regions that may be involved in monitoring symptoms.^69^
